# The distribution and correlates of self-rated health in elderly Chinese: the China Kadoorie Biobank study

**DOI:** 10.1186/s12877-019-1183-2

**Published:** 2019-06-14

**Authors:** Xingyue Song, Jing Wu, Canqing Yu, Wenhong Dong, Jun Lv, Yu Guo, Zheng Bian, Ling Yang, Yiping Chen, Zhengming Chen, An Pan, Liming Li, on behalf of the China Kadoorie Biobank Collaborative Group

**Affiliations:** 1 Department of Epidemiology and Biostatistics, and Ministry of Education Key Laboratory of Environment and Health, and State Key Laboratory of Environmental Health (Incubating), School of Public Health, Tongji Medical College, Huazhong University of Science and Technology, 13 Hangkong Rd, Wuhan, 430030 China; 2 Department of Epidemiology and Biostatistics, School of Public Health, Peking University Health Science Center, 38 Xueyuan Rd, Beijing, 100191 China; 3 Chinese Academy of Medical Sciences, Dong Cheng District, Beijing, China; 4 Clinical Trial Service Unit & Epidemiological Studies Unit (CTSU), Nuffield Department of Population Health, University of Oxford, Oxford, UK

**Keywords:** Distribution, Correlates, Self-rated health, Chinese population

## Abstract

**Background:**

Self-rated health (SRH) have been widely used as a valid indicator of health status at the population and individual level. We aimed to investigate the distribution and correlates of global SRH and age-comparative SRH in elderly Chinese.

**Methods:**

Survey of 57,693 men and 67,089 women aged 60 years and above was conducted in five rural (Gansu, Sichuan, Hunan, Henan, Zhejiang) and five urban areas (Heilongjiang, Shandong, Jiangsu, Guangxi, Hainan) in China between 2004 and 2008. Logistic regression models were used to calculate the relations of different factors with global SRH and age-comparative SRH.

**Results:**

Among the participants, 38.33% reported their global SRH as good or excellent while 61.67% as fair or poor, and 17.70% reported better age-comparative SRH while 17.99% as worse. In the multivariate model, compared to women, men tended to report a good global SRH and better age-comparative SRH, urban residents tend to report good global SRH and better age-comparative SRH. The socioeconomic and health behavior factors that were associated with good global SRH and better age-comparative SRH (with varying strengths of association) included: high educational level, high household income, house ownership, quitting smoking by own choices, occasional and current alcohol drinking, overweight, and high physical activity level. The factors that were associated with poor global SRH and worse age-comparative SRH included: quitting smoking by illness, former drinking, underweight, and weight lost ≥2.5 kg in the previous year.

**Conclusions:**

We found a moderate level of good global SRH and a low level of better age-comparative SRH among elderly Chinese. We identified a number of demographic, socioeconomic and health behavior factors that were related to SRH measures. Our study emphasizes the importance of incorporating both global and age-comparative SRH measures in future studies, and considering gender inequalities and urban/rural disparity, as well as socioeconomic status and health behaviors as important modifiers of health.

## Background

Self-rated health (SRH) is an indicator widely used to reflect a person’s general health condition and to measure health inequalities in epidemiology and public health survey. Self-health assessment is a cognitive process [1], and it is usually measured by a single question “How would you rate your health in general?”, with a three- to five-point scale from “excellent” to “very poor” [2], or by asking about “health status compared to other people of your age”, with three potential choices of “better”, “the same” and “worse” [3]. SRH is a valid predictor of mortality, disability, physical performance and frailty in older adults, and the validity has been tested in populations from Europe, Northern and South America, Oceania and most areas of Asia, including China [2, 4–6]. SRH has also been recommended by the World Health Organization as an indicator for health monitoring [7].

As the most populous country in the world, population aging has become a serious social problem in China. It is estimated that the proportion of Chinese people aged 60 and above will reach 28% by 2040 [8], and represent 65% of Chinese health burden [9]. Understanding the health status and correlates of SRH in older people is important for providing appropriate health services. Previous studies on the correlates of SRH has been extensively studied in Western populations, and reported that SRH can be affected by a range of demographic (e.g., age, gender) [10, 11], socioeconomic (e.g., marital status, education level, income) [10–12], lifestyle (e.g., smoking, alcohol, physical activity and body mass index) [10, 11, 13, 14], comorbidities and psychological factors [10, 12, 15]. Nevertheless, comprehensive assessments of the correlates of SRH among Chinese populations are lacking. Only a few studies are available, but the sample size of most studies was small and cannot represent a vast and populous country like China [16–18]. Furthermore, most existing studies focus on general global SRH, and very few studies have included the comparative (such as age-referential) SRH measure and it remains unclear whether the correlates of global and age-comparative SRH are the same or different. A previous study examined the correlates of age-comparative SRH in the Swedish population, but did not include global SRH [19]. Only one study in Finland has used both global and age-comparative SRH measures, but only investigated the relationship of age and function ability with SRH [20]. Health conditions and disease status are major correlates of SRH, as shown in many studies [3, 12, 18] and also our previous analysis [21], but more emphases should be given to the upstream social determinants of health, such as gender inequality, urban/rural disparity, socioeconomic status, and health behaviors [22].

Therefore, using data from the China Kadoorie Biobank (CKB), we aimed to investigate the distribution and correlates of good global SRH and better age-comparative SRH in elderly Chinese.

## Methods

### Study population

The CKB study is a general population-based prospective cohort study of over 0.5 million participants from 10 diverse areas across China. The detailed study design, sampling strategy and characteristics of the study participants are previously reported [23]. Briefly, a total of 512,891 participants aged 30–79 years old were recruited from five rural (Tianshui, Gansu Province; Pengzhou, Sichuan Province; Liuyang, Hunan Province; Huixian, Henan Province; Tongxiang, Zhejiang Province) and five urban areas (Harbin, Heilongjiang Province; Qingdao, Shandong Province; Suzhou, Jiangsu Province; Liuzhou, Guangxi Province; Haikou, Hainan Province) between 2004 and 2008. The study areas were selected considering a range of disease patterns and risk factors, quality of death and disease registries, and local commitment and capacity. In each study area, the subdistrict or township administrative region was designated as the investigation unit, and the potentially eligible participants selected for the study within each region were identified through official residential records, and invitation letters (with study information leaflets) were delivered door-to-door by local community leaders or health workers, following extensive publicity campaigns. As a pre-requisite for participating, all participants were asked to bring their unique national identity (ID) cards to the assessment centre set up in the local community. The current study focused on the correlates of SRH among people aged 60 years and above, thus we excluded the participants younger than 60 years (*n* = 388,108).

In the baseline survey, detailed information including general demographic characteristics, socioeconomic status, lifestyle factors, mental health and history of chronic diseases was collected by trained interviewers using a laptop-based direct data-entry system. Body height, weight, hip and waist circumference and blood pressures were measured by trained technicians.

All the participants had complete data on the variables necessary for the current analysis except for 1 participants with missing values of body mass index (BMI). Therefore, this participant was excluded and a total of 124,782 participants were available for the final analysis. The study was approved by the ethical review committee of the Chinese Center for Disease Control and Prevention (Beijing, China) and the Oxford Tropical Research Ethics Committee, University of Oxford (UK). Written informed consent forms were obtained from all participants.

### Data collection

In this study, SRH status was assessed using the following two questions: 1) How is your current general health status: excellent, good, fair, or poor? 2) How is your current health status compared with someone of your age: better, about the same, worse, or don’t know? We treated the first question as global SRH and the second one as age-comparative SRH. The global SRH was categorized into two categories in the analyses: good (excellent, good) and poor (fair, poor). We excluded the participants who answer “don’t know” (*n* = 5271, 4.22%) and “about the same” (*n* = 74,983, 60.09%) for the second question when analyzing the correlates of age-comparative SRH.

Demographic and socioeconomic factors included age, gender, study location (urban/rural), marital status (married, widowed, separated/divorced, never married), education level (no formal education, primary, middle or high school, college/university or higher), annual household income (< 1450, 1450-2899, 2890-5072, ≥ 5073 US dollars, and 1 dollar approximately equals to 6.9 Yuan), and house ownership (yes or no).

Lifestyle factors included cigarette smoking (never, former, occasionally and current smoker), alcohol consumption (never, former, occasionally, and current drinker) and weight change during the past year (unchanged, gained ≥2.5 kg and lost ≥2.5 kg). For former smokers, the main reason for cessation (already ill or stopped by choice) was also asked. The physical activity level was calculated by adding up metabolic equivalent tasks (METs) for daily work or leisure activities, and was classified into sex-specific quartiles. BMI was calculated as weight in kilograms divided by the square of height in meters and categorized according to the Chinese classification [24]: BMI < 18.5 as underweight, 18.5 ≤ BMI < 24.0 as normal weight, 24.0 ≤ BMI < 28.0 as overweight and BMI ≥28.0 as obesity.

Seven types of comorbidities including cardiometabolic diseases (diabetes, hypertension, coronary heart disease, stroke, rheumatic heart disease), respiratory diseases (tuberculosis, emphysema/bronchitis, asthma), digestive diseases (cirrhosis/chronic hepatitis, peptic ulcer, gallstone/gallbladder disease), musculoskeletal diseases (fracture, rheumatic arthritis), mental diseases (psychiatric disorders, neurasthenia, depression and generalized anxiety disorder), cancer and other diseases (kidney disease, head injury) were self-reported or measured at baseline. We have previously evaluated the relations of various comorbidities with global and age-comparative SRH measures in this cohort and the details were introduced elsewhere [21].

### Statistical analyses

Baseline characteristics by gender were presented by unadjusted means with standard deviations (SD) for continuous variables and unadjusted proportions for categorical variables, and compared using ANOVA and Chi-square tests for continuous and categorical variables, respectively. Logistic regression models were used to calculate the associations between different factors and SRH measures. The variables were adjusted in the following steps: model 1 included age, gender and study location (10 areas); model 2 additionally included social and economic indicators (marital status, education level, household income, and homeownership); model 3 further added health behaviors (smoking, alcohol, physical activity, BMI and weight change) and baseline comorbidities. We also conducted a sensitivity analysis of including participants answering “about the same” for the age-comparative SRH, and multinomial logistic regression models were used for the three-category outcome.

Stratified analyses were performed according to gender and residential area (urban and rural). Tests for interaction were conducted by adding interaction terms of the study factors and the stratifying variable in the final model. All analyses were performed using SAS 9.3 (SAS Institute Inc.), and a two-sided *P* value < 0.05 was considered as statistical significance.

## Results

### Characteristics of the participants

The baseline characteristics stratified by gender are presented in Table 1. Of the 124,782 participants, 46.24% were men and 53.76% were women. The mean age was 66.50 years for men and 66.10 years for women. Compared with women, men were more likely to be married, to have a higher education level, to be current smokers and current drinkers. A total of 38.33% reported their global SRH as “excellent” or “good” (42.15% in men and 35.06% in women, referred to “good” thereafter), and 17.70% reported better age-comparative SRH (20.40% in men and 15.37% in women).

**Table 1:** Characteristics of the study participants at baseline in the China Kadoorie Biobank study, 2004–2008^a^

Variables	Total (*n* = 124782)	Male (*n* = 57693)	Female (*n* = 67089)	*P* Value†
Global SRH status				< 0.001
Excellent	17537 (14.05)	9201 (15.95)	8336 (12.43)	
Good	30300 (24.28)	15115 (26.20)	15185 (22.63)	
Fair	60181 (48.24)	26778 (46.41)	33403 (49.79)	
Poor	16764 (13.43)	6599 (11.44)	10165 (15.15)	
Age-comparative SRH status				
Better	22083 (17.70)	11772 (20.40)	10311 (15.37)	
About the same	74983 (60.09)	35088 (60.83)	39895 (59.47)	
Worse	22445 (17.99)	8765 (15.19)	13680 (20.39)	
Don’t know	5271 (4.22)	2068 (3.58)	3203 (4.77)	
Age, mean (SD), yr	66.29 (4.37)	66.50 (4.40)	66.10 (4.33)	< 0.001
Rural residence	63771 (51.11)	31615 (54.80)	32156 (47.93)	< 0.001
Married	98555 (78.98)	50533 (87.59)	48022 (71.58)	< 0.001
Educational level				< 0.001
No formal school	40476 (32.44)	10192 (17.67)	30284 (45.15)	
Primary school	48444 (38.82)	25745 (44.62)	22699 (33.83)	
Middle or high school	29153 (23.36)	17000 (29.47)	12153 (18.11)	
College or university	6709 (5.38)	4756 (8.24)	1953 (2.91)	
Annual household income, US Dollar^b^				< 0.001
0–1449	45480 (36.45)	19696 (34.15)	25784 (38.43)	
1450–2899	35072 (28.11)	16145 (27.98)	18927 (28.21)	
2890–5072	26052 (20.88)	12590 (21.82)	13462 (20.07)	
≥ 5073	18178 (14.56)	9262 (16.05)	8916 (13.29)	
House ownership (Yes)	50160 (40.20)	23973 (41.55)	26187 (39.03)	< 0.001
Smoking status				< 0.001
Never smoker	70871 (56.80)	10853 (18.81)	60018 (89.46)	
Former smoker and quit by choices	6396 (5.12)	5585 (9.68)	811 (1.21)	
Former smoker and quit by illness	7894 (6.33)	6873 (11.91)	1021 (1.52)	
Occasional smoker	7171 (5.75)	5563 (9.64)	1608 (2.40)	
Current smoker	32450 (26.00)	28819 (49.96)	3631 (5.41)	
Alcohol status				< 0.001
Never drinker	64717 (51.86)	16939 (29.36)	47778 (71.22)	
Former drinker	4662 (3.74)	4079 (7.07)	583 (0.87)	
Occasional drinker	32130 (25.75)	16075 (27.86)	16055 (23.93)	
Current drinker	23273 (18.65)	20600 (35.71)	2673 (3.98)	
BMI, mean (SD)	23.43 (3.62)	22.94 (3.32)	23.85 (3.81)	< 0.001
Physical activity (MET-h/day), mean (SD)	13.39 (10.37)	13.33 (11.90)	13.44 (8.85)	0.08
Weight change in the past year				< 0.001
Lost ≥2.5 kg	13278 (10.64)	5895 (10.22)	7383 (11.00)	
Change ±2.5 kg	102698 (82.30)	47887 (83.00)	54811 (81.70)	
Gained ≥2.5 kg	8806 (7.06)	3911 (6.78)	4895 (7.30)	
Cardiometabolic diseases^c^ (Yes)	76650 (61.43)	34440 (59.70)	42210 (62.92)	< 0.001
Respiratory diseases^c^ (Yes)	9968 (7.99)	5768 (10.00)	4200 (6.26)	< 0.001
Musculoskeletal diseases^c^ (Yes)	13469 (10.79)	5355 (9.28)	8114 (12.09)	< 0.001
Mental diseases^c^ (Yes)	2822 (2.26)	829 (1.44)	1993 (2.97)	< 0.001
Digestives diseases^c^ (Yes)	16201 (12.98)	6799 (11.78)	9402 (14.01)	< 0.001
Cancer (Yes)	1078 (0.86)	528 (0.92)	550 (0.82)	< 0.001
Other diseases^c^ (Yes)	13085 (2.55)	550 (2.90)	6993 (2.31)	0.04

*Abbreviations*: *BMI* Body mass index, *MET* Metabolic equivalent task, *SD* Standard deviations, *SRH* Self-rated health

^a^Data are presented as frequency (percentage) unless otherwise indicated

^b^At the exchange rate as of December 2018, 1 US dollar approximately equals to 6.9 RMB Yuan

^c^Cardiometabolic diseases include diabetes, hypertension, coronary heart disease, stroke and rheumatic heart disease; Respiratory diseases include tuberculosis, emphysema/bronchitis and asthma; Digestive diseases include cirrhosis/chronic hepatitis, peptic ulcer and gallstone/gallbladder disease; Musculoskeletal diseases include fracture and rheumatic arthritis; Mental diseases include psychiatric disorders, neurasthenia, depression and generalized anxiety disorder; Other diseases include kidney disease and head injury

† Two-sided *P* values were derived from ANOVA for continuous variables and Chi-square test for categorical variables

### Factors associated with good global SRH

Table 2 shows the relations of demographic, socioeconomic factors and health behaviors with good global SRH. In the final model, the odds of reporting good global SRH was significantly higher in men and urban residents. Other factors that were associated with good global SRH included high educational level, high household income, house ownership, quitting smoking by own choices, occasional and current alcohol drinking, high level of physical activity and overweight. On the other hand, quitting smoking because of illness, former alcohol drinking, underweight and significant weight loss in the past year were associated with a lower odds of reporting good global SRH. Age and marital status were also related to global SRH, but the effect estimates were modest.

**Table 2:** Multivariate adjusted correlates of good global self-rated health status in the China Kadoorie Biobank study, 2004–2008^a^

Variables	Model 1		Model 2		Model 3^b^	
	OR	95% CI	OR	95% CI	OR	95% CI
Demographic factors						
Age (ref. = 60–64 yr)						
65–69 yr	0.91	0.88, 0.93	0.93	0.90, 0.95	1.02	0.99, 1.05
≥ 70 yr	0.84	0.81, 0.86	0.88	0.85, 0.91	1.05	1.01, 1.08
Gender (ref. = female)	1.41	1.37, 1.44	1.35	1.31, 1.38	1.26	1.22, 1.31
Residential area (ref. = Rural)	1.23	1.20, 1.25	1.20	1.17, 1.23	1.28	1.24, 1.32
Socioeconomic factors						
Marital status (ref. = Married)						
windowed			1.04	1.01, 1.07	1.06	1.02, 1.09
Separated/divorced/never married			0.84	0.75, 0.94	0.86	0.77, 0.97
Educational level (ref. = No formal school)						
Primary school			1.04	1.01, 1.08	1.05	1.01, 1.08
Middle or high school			1.13	1.09, 1.17	1.17	1.12, 1.22
College or university			1.32	1.24, 1.41	1.41	1.32, 1.50
Annual household income, US Dollar (ref. = 0–1449)						
1450–2899			1.10	1.07, 1.14	1.11	1.07, 1.14
2890–5072			1.15	1.10, 1.19	1.14	1.10, 1.19
≥ 5073			1.29	1.23, 1.34	1.29	1.23, 1.35
House ownership (ref. = No)			1.16	1.13, 1.20	1.16	1.12, 1.19
Health behaviors						
Smoking status (ref. = Never smoker)						
Former smoker and quit by choices					1.14	1.08, 1.21
Former smoker and quit by illness					0.66	0.62, 0.70
Occasional smoker					0.93	0.88, 0.98
Current smoker					0.99	0.95, 1.03
Alcohol status (ref. = Never drinker)						
Former drinker					0.80	0.75, 0.86
Occasional drinker					1.24	1.20, 1.28
Current drinker					1.41	1.36, 1.47
Physical activity (MET-h/day) (ref. = Q1)						
Q2					1.33	1.29, 1.37
Q3					1.41	1.36, 1.47
Q4					1.55	1.48, 1.63
BMI category (ref. = Normal weight)						
Underweight					0.70	0.67, 0.74
Overweight					1.10	1.07, 1.13
Obesity					0.96	0.92, 1.00
Weight change in the past year (ref. = Change ±2.5 kg)						
Lost ≥2.5 kg					0.75	0.72, 0.78
Gained ≥2.5 kg					1.08	1.03, 1.13

*Abbreviations*: *BMI* Body mass index, *CI* Confidence intervals, *MET* Metabolic equivalent task, *OR* Odds ratio

^a^For residential area, adjusted for all other variables shown in the table. For other factors, adjusted for 10 study location and all other variables shown in the table

^b^Model 3: adjusted for all other variables plus cardiometabolic diseases, respiratory diseases, musculoskeletal diseases, mental diseases, digestive diseases, cancer and other diseases

Similar findings were obtained in the stratified analyses by gender (Table 3), although the magnitude of the association varied between different strata. High educational level, high household income, occasional and current alcohol drinking, overweight and high level of physical activity was associated with good global SRH in both subgroups. The positive association between educational level and global SRH was stronger in men. Former alcohol drinking was associated with poor global SRH among men, while showed no significant association among women. Significant weight gain in the past year was associated with good global SRH among men, while showed no significant association among women. In the stratified analysis by residential area (Table 3), high educational level, high household income, occasional and current alcohol drinking, and high level of physical activity was associated with good global SRH in both subgroups. The positive association between educational level, household income and global SRH was stronger among rural residents, while the association between physical activity and global SRH was stronger among urban residents. Significant weight gain in the past year were associated with good global SRH among rural residents, but not among urban residents. Although significant interactions were found for some other variables probably because of large sample size, the effect estimates were not substantially different across strata.

**Table 3:** Multivariate adjusted correlates of good global self-rated health by residential area and gender in the China Kadoorie Biobank study, 2004–2008

Variables	Male		Female		*P* for interaction	Rural		Urban		*P* for interaction
	OR	95% CI	OR	95% CI		OR	95% CI	OR	95% CI	
Demographic factors										
Age (ref. = 60–64 yr)					0.24					0.10
65–69 yr	1.04	0.99, 1.08	1.00	0.96, 1.04		0.99	0.95, 1.03	1.06	1.02, 1.11	
≥ 70 yr	1.10	1.04, 1.15	1.00	0.95, 1.05		1.00	0.95, 1.05	1.10	1.05, 1.15	
Gender (ref. = Female)						1.21	1.15, 1.28	1.35	1.28, 1.43	0.54
Residential area (ref. = Rural)	1.26	1.21, 1.32	1.29	1.24, 1.34	0.54					
Socioeconomic factors										
Marital status (ref. = Married)					0.03					0.003
windowed	1.01	0.95, 1.07	1.08	1.04, 1.12		0.99	0.95, 1.04	1.12	1.06, 1.17	
Separated/divorced/never married	0.82	0.72, 0.94	1.08	0.85, 1.36		0.82	0.71, 0.95	0.97	0.81, 1.18	
Educational level (ref. = No formal school)					< 0.001					< 0.001
Primary School	1.01	0.96, 1.07	1.09	1.04, 1.14		1.09	1.04, 1.14	0.99	0.94, 1.04	
Middle or high School	1.17	1.10, 1.25	1.14	1.08, 1.21		1.25	1.17, 1.32	1.11	1.05, 1.18	
College or university	1.40	1.28, 1.53	1.32	1.18, 1.47		1.71	1.35, 2.17	1.37	1.26, 1.48	
Annual household income, US Dollar (ref. = 0–1449)					0.66					< 0.001
1450–2899	1.13	1.08, 1.19	1.08	1.04, 1.14		1.16	1.11, 1.21	1.03	0.98, 1.09	
2890–5072	1.17	1.11, 1.24	1.12	1.06, 1.18		1.22	1.15, 1.29	1.09	1.03, 1.15	
≥ 5073	1.36	1.27, 1.45	1.25	1.17, 1.33		1.59	1.48, 1.70	1.13	1.06, 1.20	
House ownership (ref. = No)	1.15	1.10, 1.20	1.17	1.12, 1.22	< 0.001	1.14	1.08, 1.20	1.16	1.11, 1.20	< 0.001
Health behaviors										
Smoking status (ref. = Never smoker)					0.08					< 0.001
Former smoker and quit by choices	1.13	1.05, 1.21	1.10	0.94, 1.28		1.08	0.98, 1.18	1.18	1.09, 1.28	
Former smoker and quit by illness	0.64	0.60, 0.69	0.74	0.63, 0.86		0.65	0.60, 0.71	0.67	0.61, 0.72	
Occasional smoker	0.90	0.84, 0.97	0.94	0.83, 1.05		0.94	0.87, 1.02	0.91	0.83, 0.99	
Current smoker	0.97	0.93, 1.02	1.03	0.95, 1.11		0.99	0.94, 1.05	0.99	0.93, 1.05	
Alcohol status (ref. = Never drinker)					0.001					< 0.001
Former drinker	0.81	0.74, 0.88	0.96	0.79, 1.17		0.86	0.79, 0.95	0.71	0.63, 0.79	
Occasional drinker	1.25	1.19, 1.31	1.23	1.18, 1.29		1.29	1.23, 1.35	1.20	1.15, 1.26	
Current drinker	1.42	1.35, 1.48	1.56	1.43, 1.70		1.43	1.36, 1.51	1.37	1.30, 1.45	
Physical activity (MET-h/day) (ref. = Q1)					0.07					< 0.001
Q2	1.35	1.29, 1.41	1.34	1.29, 1.39		1.32	1.26, 1.37	1.35	1.30, 1.41	
Q3	1.42	1.34, 1.51	1.40	1.33, 1.48		1.25	1.19, 1.32	1.72	1.61, 1.83	
Q4	1.71	1.60, 1.84	1.42	1.32, 1.52		1.35	1.27, 1.43	2.17	1.96, 2.39	
BMI category (ref. = Normal weight)					0.18					0.13
Underweight	0.67	0.63, 0.72	0.72	0.67, 0.78		0.74	0.69, 0.78	0.64	0.58, 0.71	
Overweight	1.11	1.06, 1.16	1.09	1.05, 1.14		1.07	1.03, 1.12	1.13	1.09, 1.18	
Obesity	0.99	0.92, 1.07	0.94	0.89, 0.99		0.90	0.84, 0.97	1.01	0.95, 1.06	
Weight change in the past year (ref. = Change ±2.5 kg)					0.10					< 0.001
Lost ≥2.5 kg	0.72	0.67, 0.76	0.77	0.73, 0.81		0.69	0.65, 0.73	0.79	0.75, 0.84	
Gained ≥2.5 kg	1.11	1.04, 1.19	1.05	0.98, 1.12		1.15	1.07, 1.23	1.02	0.96, 1.09	

Odds ratios (95% CI) were calculated after adjustment of other variables shown in the table plus 10 study locations, cardiometabolic diseases, respiratory diseases, musculoskeletal diseases, mental diseases, digestive diseases, cancer and other diseases, except for the stratified variable in the corresponding stratified analysis

*Abbreviations*: *BMI* Body mass index, *CI* Confidence intervals, *MET* Metabolic equivalent task, *OR* Odds ratio

### Factors associated with better age-comparative SRH

Table 4 shows the results for better age-comparative SRH in the total study population. In the final model, the odds of reporting better age-comparative SRH was significantly higher in men, urban residents and older individuals. Other factors that were associated with better age-comparative SRH included being windowed, high educational level, high household income, house ownership, quitting smoking by own choices, occasional and current alcohol drinking, high level of physical activity and overweight. The factors that associated with worse age-comparative SRH included quitting smoking because of illness, former alcohol drinking, underweight and significant weight loss.

**Table 4:** Multivariate adjusted correlates of better age-comparative self-rated health status in the China Kadoorie Biobank study, 2004–2008^a^

Variables	Model 1		Model 2		Model 3^b^	
	OR	95% CI	OR	95% CI	OR	95% CI
Demographic factors						
Age (ref. = 60–64 yr)						
65–69 yr	0.92	0.88, 0.97	0.99	0.95, 1.04	1.21	1.15, 1.27
≥ 70 yr	0.97	0.92, 1.02	1.12	1.06, 1.18	1.63	1.54, 1.73
Gender (ref. = Female)	1.90	1.83, 1.98	1.65	1.58, 1.73	1.43	1.33, 1.53
Residential area (ref. = Rural)	2.19	2.11, 2.28	1.70	1.63, 1.78	1.93	1.84, 2.03
Socioeconomic factors						
Marital status (ref. = Married)						
windowed			1.15	1.09, 1.21	1.20	1.13, 1.26
Separated/divorced/never married			0.85	0.70, 1.03	0.95	0.77, 1.16
Educational level (ref. = No formal school)						
Primary school			1.24	1.18, 1.31	1.27	1.20, 1.35
Middle or high school			1.70	1.59, 1.82	1.84	1.72, 1.98
College or university			2.36	2.12, 2.64	2.76	2.45, 3.12
Annual household income, US Dollar (ref. = 0–1449)						
1450–2899			1.20	1.14, 1.27	1.21	1.14, 1.28
2890–5072			1.34	1.25, 1.43	1.35	1.26, 1.45
≥ 5073			1.56	1.44, 1.68	1.58	1.45, 1.71
House ownership (ref. = No)			1.28	1.28, 1.35	1.30	1.23, 1.37
Health behaviors						
Smoking status (ref. = Never smoker)						
Former smoker and quit by choices					1.32	1.18, 1.48
Former smoker and quit by illness					0.50	0.45, 0.55
Occasional smoker					1.01	0.92, 1.12
Current smoker					1.05	0.98, 1.13
Alcohol status (ref. = Never drinker)						
Former drinker					0.67	0.59, 0.75
Occasional drinker					1.62	1.53, 1.71
Current drinker					2.13	1.99, 2.28
Physical activity (MET-h/day) (ref. = Q1)						
Q2					1.78	1.69, 1.88
Q3					2.36	2.20, 2.52
Q4					2.77	2.53, 3.04
BMI category (ref. = Normal weight)						
Underweight					0.49	0.45, 0.54
Overweight					1.30	1.24, 1.37
Obesity					0.99	0.92, 1.07
Weight change in the past year (ref. = Change ±2.5 kg)						
Lost ≥2.5 kg					0.57	0.54, 0.61
Gained ≥2.5 kg					1.04	0.96, 1.12

*Abbreviations*: *BMI* Body mass index, *CI* Confidence intervals, *MET* Metabolic equivalent task, *OR* Odds ratio

^a^Participants answering “don’t know” (*n* = 5271) and “about the same” (*n* = 74,983) for the age-comparative self-rated health status question were excluded from the analysis, leaving 44,528 participants in this analysis. For residential area, adjusted for all other variables shown in the table. For other factors, adjusted for 10 study location and all other variables shown in the table

^b^Model 3: adjusted for all other variables plus cardiometabolic diseases, respiratory diseases, musculoskeletal diseases, mental diseases, digestive diseases, cancer and other diseases

In the stratified analyses by gender (Table 5), older age, high household income, occasional and current alcohol drinking, high level of physical activity, overweight was associated with better age-comparative SRH in both subgroups. The positive association between age, household income, overweight and age-comparative SRH was stronger in men. Former alcohol drinking was associated with worse age-comparative SRH in men, but not in women. In the stratified analysis by residential area (Table 5), high educational level, high household income, occasional and current alcohol drinking, high level of physical activity and overweight was associated with better age-comparative SRH in both subgroups. The positive association between educational level, household income and age-comparative SRH was stronger among rural residents, while the association between physical activity and age-comparative SRH was stronger among urban residents. Significant weight gain in the past year were associated with better age-comparative SRH among rural residents, while show opposite association among urban residents. Although significant interactions were found for some other variables, the effect estimates were not substantially different.

**Table 5:** Multivariate adjusted correlates of better age-comparative self-rated health status by residential area and gender in the China Kadoorie Biobank study, 2004–2008^a^

Variables	Male		Female		*P* for interaction	Rural		Urban		*P* for interaction
	OR	95% CI	OR	95% CI		OR	95% CI	OR	95% CI	
Demographic factors										
Age (ref. = 60–64 yr)					0.03					0.64
65–69 yr	1.25	1.16, 1.36	1.18	1.10, 1.26		1.17	1.09, 1.27	1.25	1.16, 1.35	
≥ 70 yr	1.88	1.72, 2.06	1.45	1.34, 1.57		1.60	1.46, 1.74	1.67	1.54, 1.81	
Gender (ref. = Female)						1.35	1.22, 1.48	1.55	1.40, 1.70	0.02
Residential area (ref. = Rural)	1.69	1.56, 1.83	2.13	2.00, 2.28	0.02					
Socioeconomic factors										
Marital status (ref. = Married)					0.05					0.009
windowed	1.07	0.96, 1.19	1.24	1.16, 1.32		1.09	1.00, 1.17	1.30	1.20, 1.42	
Separated/divorced/never married	0.93	0.72, 1.18	1.05	0.72, 1.55		0.79	0.60, 1.05	1.21	0.89, 1.65	
Educational level (ref. = No formal school)					0.01					< 0.001
Primary School	1.27	1.16, 1.40	1.29	1.20, 1.39		1.32	1.22, 1.42	1.21	1.11, 1.32	
Middle or high School	1.98	1.77, 2.22	1.68	1.53, 1.85		2.06	1.84, 2.29	1.70	1.54, 1.88	
College or university	2.75	2.34, 3.25	2.77	2.30, 3.34		3.23	2.15, 4.84	2.60	2.26, 2.99	
Annual household income, US Dollar (ref. = 0–1449)					0.02					< 0.001
1450–2899	1.30	1.19, 1.42	1.15	1.07, 1.24		1.22	1.13, 1.33	1.18	1.08, 1.29	
2890–5072	1.50	1.34, 1.67	1.28	1.17, 1.41		1.52	1.34, 1.73	1.32	1.20, 1.45	
≥ 5073	1.91	1.68, 2.17	1.38	1.24, 1.55		1.98	1.68, 2.34	1.50	1.35, 1.67	
House ownership (ref. = No)	1.25	1.15, 1.36	1.33	1.24, 1.43	0.03	1.24	1.14, 1.36	1.31	1.22, 1.40	< 0.001
Health behaviors										
Smoking status (ref. = Never smoker)					< 0.001					0.43
Former smoker and quit by choices	1.32	1.15, 1.51	1.06	0.84, 1.35		1.40	1.17, 1.67	1.27	1.10, 1.47	
Former smoker and quit by illness	0.45	0.40, 0.51	0.64	0.52, 0.80		0.52	0.45, 0.61	0.48	0.42, 0.55	
Occasional smoker	0.98	0.86, 1.12	0.90	0.75, 1.07		1.07	0.94, 1.22	0.96	0.82, 1.12	
Current smoker	1.01	0.92, 1.11	1.04	0.93, 1.18		1.09	0.99, 1.20	1.01	0.91, 1.12	
Alcohol status (ref. = Never drinker)					< 0.001					< 0.001
Former drinker	0.63	0.55, 0.72	1.21	0.92, 1.60		0.82	0.70, 0.96	0.51	0.42, 0.61	
Occasional drinker	1.73	1.58, 1.89	1.53	1.43, 1.65		1.64	1.52, 1.78	1.62	1.50, 1.75	
Current drinker	2.19	2.01, 2.40	2.50	2.16, 2.89		2.27	2.05, 2.50	2.00	1.81, 2.21	
Physical activity (MET-h/day) (ref. = Q1)					0.06					< 0.001
Q2	1.87	1.72, 2.03	1.75	1.63, 1.87		1.69	1.56, 1.83	1.85	1.72, 1.98	
Q3	2.38	2.14, 2.65	2.29	2.09, 2.50		2.12	1.93, 2.32	2.72	2.44, 3.03	
Q4	3.33	2.89, 3.83	2.38	2.10, 2.70		2.29	2.04, 2.58	4.06	3.42, 4.81	
BMI category (ref. = Normal weight)					0.003					0.01
Underweight	0.47	0.42, 0.54	0.50	0.44, 0.57		0.56	0.50, 0.63	0.40	0.34, 0.47	
Overweight	1.45	1.34, 1.57	1.20	1.12, 1.28		1.19	1.10, 1.28	1.39	1.30, 1.49	
Obesity	1.14	1.00, 1.29	0.91	0.83, 0.99		0.90	0.79, 1.02	1.07	0.97, 1.17	
Weight change in the past year (ref. = Change ±2.5 kg)					0.002					< 0.001
Lost ≥2.5 kg	0.53	0.48, 0.59	0.61	0.56, 0.66		0.55	0.50, 0.61	0.59	0.54, 0.64	
Gained ≥2.5 kg	1.15	1.02, 1.30	0.96	0.87, 1.06		1.31	1.17, 1.48	0.86	0.78, 0.96	

Odds ratios (95% CI) were calculated after adjustment of other variables shown in the table plus 10 study locations, cardiometabolic diseases, respiratory diseases, musculoskeletal diseases, mental diseases, digestive diseases, cancer and other diseases, except for the stratified variable in the corresponding stratified analysis

*Abbreviations*: *BMI* Body mass index, *CI* Confidence intervals, *MET* Metabolic equivalent task, *OR* Odds ratio

^a^Participants answering “don’t know” (*n* = 5271) and “about the same” (*n* = 74,983) for the age-comparative self-rated health status question were excluded from the analysis, leaving 44,528 participants in this analysis

In the sensitivity analysis of including participants who answered “about the same” for the age-comparative SRH, the results did not change materially (Table 6 in Appendix).

## Discussion

In this large population-based study, we found a moderate level of good global SRH (38.33%) and a low level of better age-comparative SRH (17.70%) among elderly Chinese. In general, men and urban residents were more likely to report good/better SRH compared to women and rural residents. People with high socioeconomic status (education level, household income and house ownership) and health behaviors (physical activity, low to moderate alcohol consumption, quitting smoking by own choices) were more likely to report good/better SRH, while people with underweight or significant weight loss in the past year were more likely to report poor/worse SRH. Age was positively associated with age-comparative SRH, indicating a survival advantage.

Some previous studies have reported large variations in the level of good/excellent global SRH in Chinese populations, ranging from 25.2 to 47% in different studies [6, 16, 18, 25, 26]. As noted, the global SRH is highly influenced by the characteristics of the study population (age, gender composition, urban/rural, health status etc.) and large variations are expected due to the different selection criteria in various studies. Therefore, direct comparison of our study results to those previous reports in the Chinese populations may not be meaningful. Most prior studies were restricted to subpopulations in one or two certain areas or institutions, and the sample size ranged from 411 to 12,583. Our study is possibly the largest population-based study from 10 diverse regions of China focusing on correlates of SRH. Our prevalence estimate (38.33% reporting good/excellent global SRH) was comparable to a previous study among 1433 participants aged 60 years and above in one urban and two rural areas of Beijing and Shanghai (38.1%) [6].

Most participants assessed their age-comparative SRH as “about the same” (60.09%), 17.70% as “better” and 17.99% as “worse”. Similar levels of age-comparative SRH were found in a study among 62,824 residents aged ≥65 years in Hong Kong (72.9% as “about the same” and 25.3% as “better”) [5]. However, a study in Finland found a level of similar age-comparative SRH of 28% and a level of better age-comparative SRH of 42% [27]. The Chinese “doctrine of the mean” advocates modesty which may make people more likely to rate their age-comparative SRH as “about the same” or “similar”. Again, the selection criteria of the study population may also have a big influence.

Among the demographic variables, we found that older people were more likely to report better age-comparative SRH but similar global SRH, the results of age-comparative SRH were consistent with previous studies, but the results of global SRH were not entirely consistent with previous studies [3, 19, 20]. When reporting global SRH, people may compare their current health status with that of their younger ages or with other younger people, but in the context of age-comparative SRH, they compared their health status with someone of similar ages. Survival bias is a possible explanation for better age-comparative SRH related to old age because those elderly people who participated in the study could have generally good health status compared to those who could not participate (e.g., because of premature death or severe diseases). Previous studies indicated that elderly people tended to overestimate their own heath or underestimate the health of others of their ages, and there may be a deterioration of judgment with increasing age or a healthy survivor effect [3, 20]. In addition, the perception of health status could be different in old people, the continuous reduction in the level of health expectations allows older people to better adapt to the aging process. We also found that men tended to report a good global SRH and better age-comparative SRH compared with women, which was consistent with most previous studies [12, 19]. Gender differences in SRH may be due to the differences in social status, social stress, family roles, pain tolerance and health expectations between men and women [28]. Men in our study were more likely to have a higher education level, although we have controlled for education level in the models, residual confounding of socioeconomic status is still possible and we could not adequately adjust for other aspects of the socioeconomic inequalities between men and women. In addition, compared with women, men are more tolerant of various physical pains and more optimistic about health, and the gender differences in mental health status (e.g., women reporting higher probability of depression) have been well-established [29]. Urban residents were more likely to report good/better SRH status in our study. The rural residents in our study were mostly engaged in agricultural work, had low social status and low income, and there were dramatic differences in living environment and medical conditions between urban and rural areas. Taken together, our study cannot fully explain the exact reasons for the gender and urban/rural differences in reporting health status, but since gender inequality and urban/rural health disparity are generally upstream determinants of socioeconomic status and health behaviors, our results further emphasize the importance of establishing social policy to achieve greater social and economic equality in society in order to reduce the health inequity.

The relations of marital status with SRH were not consistently reported in the literature [12, 18, 19], and we found that the associations were generally null or modest. Consistent with previous studies [16–19], we found that people with higher educational level and household income levels were more likely to report better global and age-comparative SRH. People with higher level of education and income may have better living conditions, better recognition of healthy lifestyles, and greater ability to withstand health risks and control their own health. Therefore, our results echo the recommendations from the WHO Commission on Social Determinants of Health that more actions are needed to tackle the health disparity by focusing on social determinants [22].

As for health behaviors, we found that healthy lifestyles (physical activity and low to moderate alcohol consumption) were associated with both good global SRH and better age-comparative SRH, although the associations were generally stronger for age-comparative SRH. We did not find significant association with current smoking, while the association with past smoking depended on the reason of quitting smoking. People who quitted smoking because of illness were less likely to report good SRH status, which may reflect the fact that they were in poor physical health status. In our study, people who quitted smoking by their own choices were more likely to report good SRH status, which may be because those individuals paid more attention to their lifestyles and health status. Current drinking was significantly associated with better SRH in our study, particularly for age-comparative SRH, which is consistent with another study in China [18]. The reason may be that regular drinker have formed a suitable amount of drinking over the lifetime, and this lifestyle can bring social benefits, pleasure and relieve fatigue. It should be noted that most of the current drinkers drank low-to-moderate amount of alcohol, which has been shown to be part of healthy lifestyles and associated with better health outcomes, including healthy ageing [30]. On the contrast, former alcohol drinkers were less likely to report good SRH, and it is possible that many people stopped drinking because of physical health conditions. Consistent with previous studies [18, 19, 31], we also found that high levels of physical activity were associated with good SRH status. Regular physical activity can help improve physical and mental function as well as alleviate the adverse effects of some chronic diseases [32], and physical activity is positively associated with healthy ageing [33].

In our study, underweight was significantly associated with poor global SRH and worse age-comparative SRH, consistent with previous studies [14, 34]. On the other hand, overweight was associated with better SRH, particularly for age-comparative SRH, which was not in agreement with previous studies in Caucasians [14, 19, 35]. Many Chinese do not consider overweight/obesity as a disease but as a symbol of wealth [36]. Previous studies in China also found an inverse association between overweight and obesity and depression [37, 38], and indicated that overweight/obesity people may be more optimistic about life and health, like a saying: “laughing and growing fat is a blessing”. When we further examined the impact of weight change on SRH, we found people who lost more than 2.5 kg weight in the past year were less likely to report both good global SRH and better age-comparative SRH, which may be due to disease-induced weight loss. Meanwhile, people who gained weight more than 2.5 kg were more likely to report a good global SRH but show no significant association with better age-comparative SRH.

The relations of most variables with the two SRH measures in different subgroups were in the same direction, but the effect sizes varied substantially. We found that socioeconomic status (education level, household income) and health behaviors (smoking and drinking status) had a stronger influence on global SRH and age-comparative SRH in men. Furthermore, socioeconomic status (educational level and household income) had a stronger influence on global SRH and age-comparative SRH among rural residents, and health behaviors (physical activity) had a stronger influence on global SRH and age-comparative SRH among urban residents. The results further confirm the impact of gender inequality and urban-rural economic differences on health, and emphasize the importance of healthy lifestyle to improve personal health among urban residents.

For the correlates of the two SRH measures, our study further confirmed that older people tended to report better age-comparative SRH. Overall, the relations of different variables with the two SRH measures were in the same direction, but the effect sizes varied substantially. For example, the associations were generally stronger for age-comparative SRH with socioeconomic status (education level and household income), health behaviors (alcohol intake, physical activity, BMI and weight change). Although the exact mechanisms are unclear, our study indicated that future studies should incorporate both global and age-comparative SRH to provide complementary information.

The strength of this study is that we used both global and age-comparative SRH measures as outcomes and the analyses covered a wide range of demographic, socioeconomic, and lifestyle factors. The study was based on a large and diverse population that provided us sufficient power to detect modest associations. However, several limitations should be noted. First, the study participants were not selected to represent the whole China, and the generalizability of the study findings should be cautious despite the large sample size. We did not include people aged 80 years and older, and again the generalizability to much older populations is unclear given that the age group over 80 years comprises large proportion of people with disability and poor health. However, our major findings of gender inequalities, urban/rural health divide, socioeconomic status and health behaviors as major correlates of health should still be valid and have important policy implications for the current Chinese society, and even for other populations undergoing nutrition and health transitions. Second, many variables were self-reported and the findings may be subject to residual confounding and recall bias if people with different SRH levels recalled things differently, although the participants were face-to-face interviewed by well-trained interviewers. Third, because of the large sample size, even modest effect size could be statistically significant, including the interaction tests; therefore, the results should be interpreted cautiously and we only discussed variables that showed relatively strong associations with the outcomes. Fourth, several factors which have been found to be associated with SRH in previous studies, such as functional ability, cognitive levels and social networks [20, 34], were not available in this study. Finally, causal relations cannot be determined in a cross-sectional study like ours.

## Conclusions

We found a moderate level of good global SRH and a low level of better age-comparative SRH among elderly Chinese. We identified a number of demographic, socioeconomic and health behaviors that were significantly associated with global and age-comparative SRH, with varying degrees and directions of effect estimates. Therefore, future studies should incorporate both global and age-comparative SRH to provide complementary information on the health status of the individuals and population. The significant differences between men and women, urban and rural residents in reporting SRH and the different correlates of SRH also indicate the importance of considering gender and urban/rural inequalities as the upstream determinants of health status. In addition, the consistent and strong impact of high socioeconomic status on SRH also provides evidence for reducing health inequity through social policy to achieve greater social and economic equality in society. Finally, our study also highlights the potential of improving personal and population health by modifiable behavior factors that include smoking cessation and regular exercise.

## Data Availability

The data that support the findings of this article are available from the CKB upon reasonable request (http://www.ckbiobank.org/site/Data+Access).

## Appendix

**Table 6:** Correlates of better age-comparative self-rated health status in the China Kadoorie Biobank study, 2004–2008: multinomial logistic regression analysis^a^

Variables	Worse	About the same		Better	
	OR	OR	95% CI	OR	95% CI
Demographic factors					
Age (ref. = 60–64 yr)					
65–69 yr	1	1.06	1.02, 1.10	1.20	1.15, 1.26
≥ 70 yr	1	1.28	1.23, 1.34	1.62	1.53, 1.71
Sex (ref. = Women)	1	1.23	1.17, 1.29	1.43	1.34, 1.52
Residential area (ref. = Rural)	1	1.08	1.04, 1.12	1.97	1.88, 2.07
Socioeconomic factors					
Marital status (ref. = Married)					
windowed	1	1.05	1.00, 1.09	1.19	1.13, 1.25
Separated/divorced/never married	1	0.85	0.74, 0.97	0.89	0.74, 1.08
Educational level (ref. = No formal school)					
Primary school	1	1.22	1.17, 1.27	1.27	1.20, 1.34
Middle or high school	1	1.33	1.26, 1.41	1.84	1.73, 1.97
College or university	1	1.60	1.45, 1.77	2.66	2.38, 2.98
Annual household income, US Dollar (ref. = 0–1449)					
1450–2899	1	1.17	1.12, 1.22	1.21	1.14, 1.27
2890–5072	1	1.30	1.24, 1.37	1.36	1.28, 1.46
≥ 5073	1	1.32	1.25, 1.41	1.63	1.51, 1.76
House ownership (ref. = No)	1	1.13	1.09, 1.18	1.32	1.26, 1.39
Health behaviors					
Smoking status (ref. = Never smoker)					
Former smoker and quit by choices	1	1.16	1.06, 1.27	1.29	1.17, 1.43
Former smoker and quit by illness	1	0.59	0.55, 0.64	0.50	0.45, 0.55
Occasional smoker	1	0.98	0.90, 1.06	0.98	0.89, 1.08
Current smoker	1	0.98	0.93, 1.04	1.04	0.97, 1.11
Alcohol status (ref. = Never drinker)					
Former drinker	1	0.68	0.63, 0.73	0.68	0.61, 0.77
Occasional drinker	1	1.38	1.32, 1.44	1.61	1.53, 1.70
Current drinker	1	1.60	1.52, 1.69	2.15	2.01, 2.30
Physical activity (MET-h/day) (ref. = Q1)					
Q2	1	1.48	1.42, 1.54	1.84	1.75, 1.93
Q3	1	1.78	1.69, 1.87	2.53	2.38, 2.70
Q4	1	2.10	1.96, 2.25	2.97	2.73, 3.24
BMI category (ref. = Normal weight)					
Underweight	1	0.59	0.56, 0.63	0.49	0.45, 0.54
Overweight	1	1.17	1.13, 1.22	1.28	1.22, 1.34
Obesity	1	1.00	0.95, 1.05	1.02	0.95, 1.09
Weight change in the past year (ref. = Change ±2.5 kg)					
Lost ≥2.5 kg	1	0.54	0.52, 0.57	0.55	0.52, 0.58
Gained ≥2.5 kg	1	0.80	0.75, 0.85	1.00	0.93, 1.08

For residential area, adjusted for all other variables plus cardiometabolic diseases, respiratory diseases, musculoskeletal diseases, mental diseases, digestive diseases, cancer and other diseases. For other factors, adjusted for 10 study location, all other variables shown in the table, and plus cardiometabolic diseases, respiratory diseases, musculoskeletal diseases, mental diseases, digestive diseases, cancer and other diseases

*Abbreviations*: *BMI* Body mass index, *CI* Confidence intervals, *MET* Metabolic equivalent task, *OR* Odds ratio

^a^Participants answering “don’t know” (*n* = 5271) for the age-comparative self-rated health status question were excluded from the analysis, leaving 119,511 participants in this analysis

## Abbreviations

BMI: Body mass index
CI: Confidence intervals
CIDI-SF: Composite International Diagnostic Inventory-short form
CKB: China Kadoorie Biobank
METs: Metabolic equivalent tasks
OR: Odds ratio
SRH: Self-rated health
